# Lateralizing Calcaneal Osteotomy and First Metatarsal Dorsiflexion Osteotomy for Cavovarus Foot and Peroneal Sheath Release with Peroneus Brevis Repair for Peroneal Tendinopathy in Chronic Ankle Instability and Sprain

**DOI:** 10.7759/cureus.32235

**Published:** 2022-12-05

**Authors:** Kazuki Kisamori, Tadashi Kimura, Mitsuru Saito, Makoto Kubota

**Affiliations:** 1 Department of Orthopaedic Surgery, Jikei University School of Medicine, Tokyo, JPN

**Keywords:** tubulization, first metatarsal osteotomy, calcaneal osteotomy, peroneal tendinopathy, pes cavus

## Abstract

A 47-year-old male presented with an eight-year history of pain in the posterior inferior part of the lateral malleolus, ankle instability, and repeated right-sided ankle sprains. He had pes cavus and hind-foot varus in his right foot, which is an unknown congenital entity or acquired with tenderness in the inferior peroneal retinaculum. There is no deformity in his left foot. The pain was elicited by the movement of the subtalar joint. Imaging revealed a high medial longitudinal arch, an enlarged peroneal tubercle, thinning of the peroneus brevis tendon, and hypertrophy of the peroneus longus tendon. We diagnosed peroneal tendinopathy with cavovarus foot in a chronic ankle sprain. The supination generated by pes cavus was thought to be aggravating the peroneal tendinopathy and causing the ankle sprains. Incision of the peroneal tendon sheath, repair of the peroneus brevis tendon, lateralizing calcaneal osteotomy, and first metatarsal dorsiflexion osteotomy were performed. At the one-year follow-up, Meary's angle was corrected to 0°, the calcaneal pitch was corrected to 20°, and the hindfoot varus was improved. He was pain-free and reported no further instability when walking. His Japanese Society of Surgery of the Foot ankle-hindfoot scale score improved from 59 preoperatively to a maximum of 100 and the Self-Administered Foot Evaluation Questionnaire gave an almost perfect score for non-sports-related items and a score of 83.3 for sports-related items. We believe that the addition of treatment of the pes cavus, which was the center of the pathology, as well as treatment of the peroneal tendon, resulted in a good outcome.

## Introduction

Peroneal tendinopathy was first described by Meyer in 1924 [1]. It causes pain in the lateral aspect of the hindfoot, and if conservative treatments, such as rest and medication, are not effective, surgical treatment, such as tendon sheath incision or tendon repair, is the next treatment. However, anatomical abnormalities, such as a giant peroneal tubercle, the presence of a fourth peroneal muscle, the presence of os peroneum, chronic ankle instability, and pes cavus have been identified to cause peroneal tendinopathy and should also be treated when present [2]. We report a case in which an additional procedure was conducted to improve the pes cavus, which resulted in a good outcome.

## Case presentation

**Figure 1:**
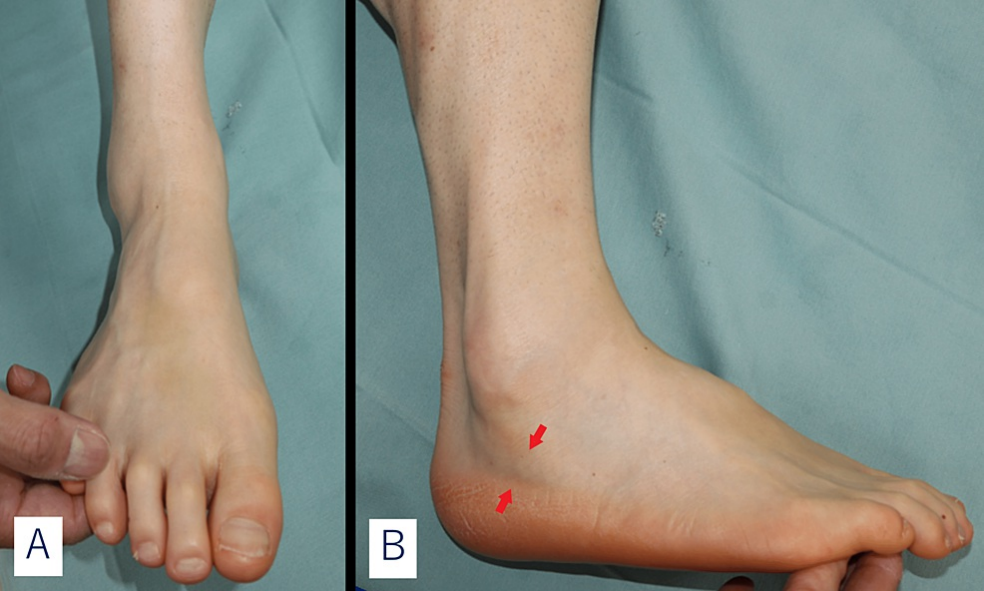
Pes cavus in the right foot. (A) Dorsoplantar view. (B) There was swelling and tenderness at the inferior peroneal retinaculum(arrow).

The patient was a 47-year-old male physical therapist. Although he enjoyed jogging, he had been experiencing pain in the posterior inferior part of the lateral malleolus of the right ankle and ankle instability in the right as his chief complaint for the previous eight years. He also had a history of repeated ankle sprains, an avulsion fracture of the distal fibula, and a fracture of the fifth metatarsus. He had been diagnosed with lateral ankle ligament insufficiency and peroneal tendinopathy by another doctor, who suggested lateral ligament reconstruction and tendon sheath incision. He visited our department for a second opinion. There was swelling and tenderness in the inferior peroneal retinaculum in the right foot, and pain was elicited in the same area by passive maximum inversion and in the subtalar joint from the eversion position on resistance exercise. There was no limitation in the range of motion of the ankle joint. Instability of the ankle joint was observed but was not significant. Furthermore, he had a pes cavus, a medial longitudinal arch that was higher than the lateral one, resulting in hindfoot varus (Figure 1).

**Figure 2:**
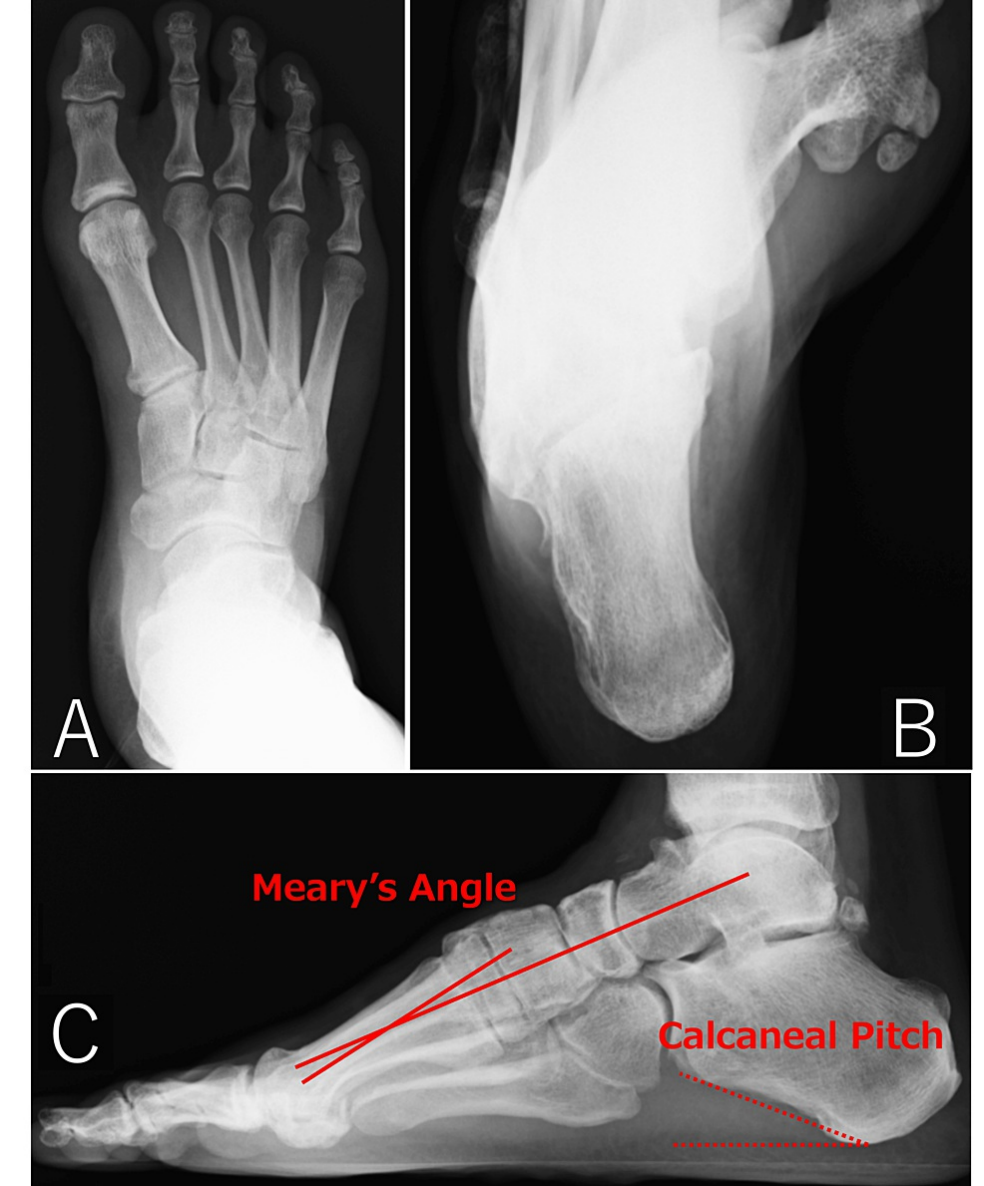
Plain weight-bearing radiographs (A) Dorsoplantar view. The avulsion fracture of the distal fibula and the fracture of the fifth metatarsal, which are the patient's post injuries are not seen in the images. (B) Non-weight bearing axial view of the calcaneus. Marked varus was seen at the right calcaneus. (C) Lateral view. Meary’s angle was 13° upward convex, the calcaneal pitch was 23°, and the navicular bone was elevated markedly above the floor.

There was no sensory disturbance in either area. On plain radiographs, Meary’s angle (the angle between a line drawn along the lateral longitudinal axes of the talus and the first metatarsal. -4° - +4° is normal) was 13° convex upward with a calcaneal pitch of 23° (10°-25° is normal), the navicular was markedly elevated above the floor, there was a pes cavus characterized by the elevation of the medial longitudinal arch, and the varus in the calcaneal axial projection was more marked than on the left side (Figure 2).

**Figure 3:**
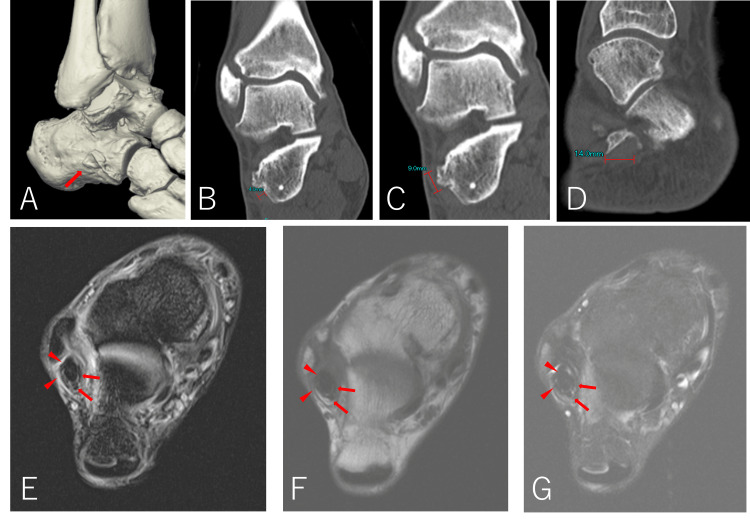
Preoperative images (A-D) Three-dimensional and plane computed tomography scan showing a 9 × 14 × 4 mm osteophyte at the peroneal tubercle(arrow). (E) T1-weighted, (F) T2-weighted, and (G) Short tau inversion recovery axial magnetic resonance image showing a thinned peroneus brevis tendon (arrow) and a hypertrophied peroneus longus tendon (arrowhead).

Computed tomography scans showed a 9 × 14 × 4 mm osteophyte at the peroneal tubercle, and the formation of osteophytes on the anterior aspects of the lower end of the tibia and talus and tarsal coalescence was not observed (Figure 3A-D). T2-weighted magnetic resonance images showed thinning of the peroneus brevis tendon with internal high-signal areas, a hypertrophic peroneus longus tendon (Figure 3E-G), and bone marrow edema confined to the medial side of the tibial plafond. The patient was diagnosed with peroneal tendon disorder at the inferior peroneal retinaculum, and the ankle supination caused by pes cavus was considered to be aggravating the peroneal tendon disorder and causing the ankle sprains. Given the patient’s history of multiple sprains and fractures, surgery was considered necessary to correct the pes cavus and treat the peroneal tendon to obtain an acceptable level of improvement.

Treatment

**Figure 4:**
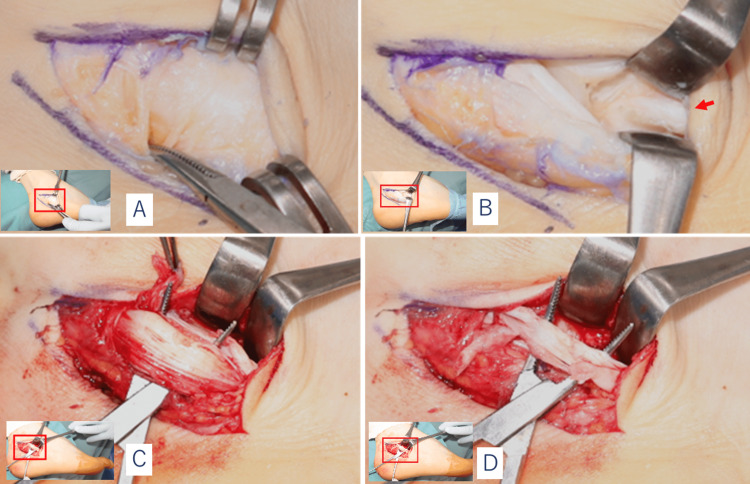
Intraoperative images (A) Thickened tendon sheath. (B) Enlarged peroneal tubercle (septum of the tendon sheath) (arrow). (C) Markedly hypertrophied peroneus longus tendon. There was no low-lying muscle belly. (D) Partially ruptured and thinned peroneal brevis tendon.

Under general anesthesia, the patient was placed in the lateral recumbent position and the operation was started with a tourniquet applied to the thigh. A 3-cm skin incision was made superiorly, anterior to the medial malleolus, the inside of the ankle joint was observed, and the anterior osteophyte was resected. And a 7-cm arcuate skin incision was made along the inferior border of the peroneal tendon from the inferior end of the lateral malleolus to the calcaneocuboid joint. The common sheath of the long and short peroneal tendon within the inferior peroneal retinaculum was found to be significantly thickened (Figure 4A). Although both tendons remained continuous, the peroneal brevis tendon was partially ruptured and thin (Figure 4D) and the peroneus longus tendon was markedly hypertrophied (Figure 4C). When the tendon was moved off, an enlarged septum was seen between the two tendon sheaths (Figure 4B), which corresponded to the osteophyte of the peroneal tubercle seen in the images. After the excision of the osteophyte, the partial tear of the peroneus brevis tendon was repaired by tubulization sutures using nylon thread. Next, the lateral aspect of the calcaneus was accessed via the same skin incision, and a lateralizing calcaneal osteotomy was performed.

**Figure 5:**
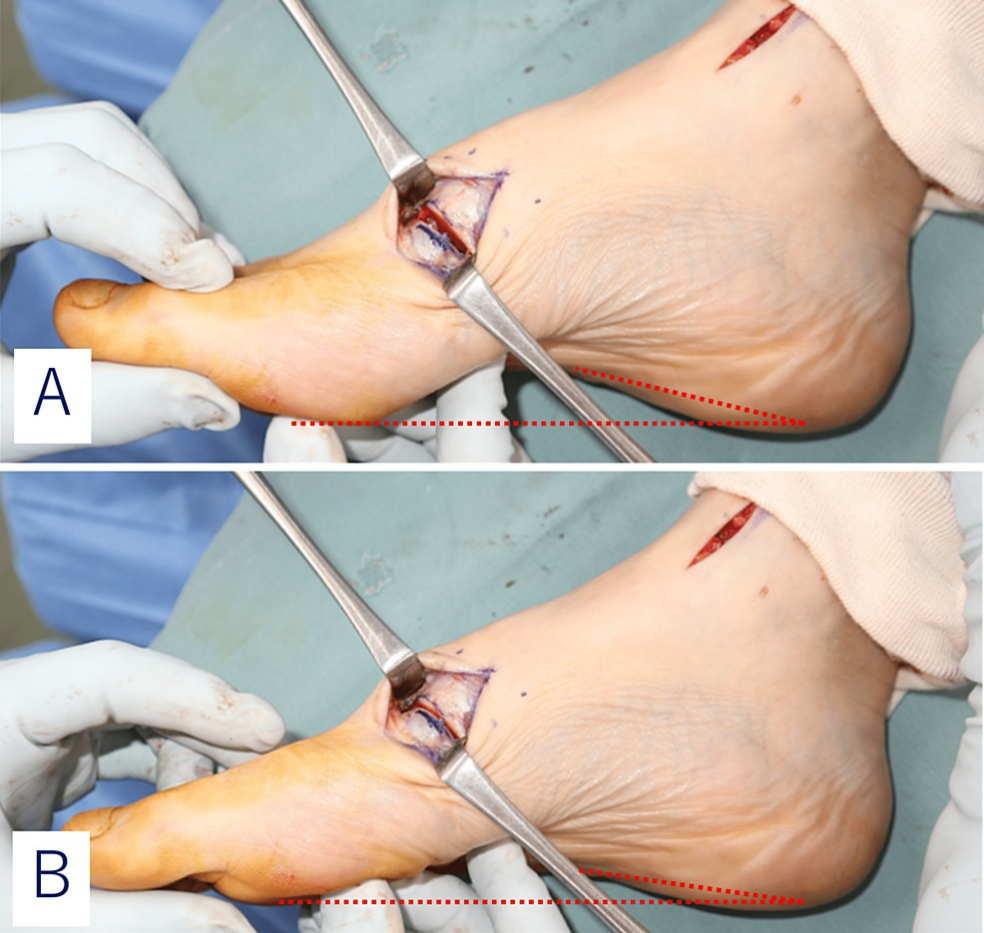
Dorsiflexion osteotomy at the first metatarsal (A) The dorsal resection width was 5 mm. (B) After dorsiflexion, the medial longitudinal arch was flattened. The upper wound was used to resect an anterior ankle joint osteophyte.

An osteotomy was made at the body of the calcaneus using a bone saw, and the posterior part of the calcaneal tuberosity was moved 7 mm laterally and fixed with a single headless screw. Finally, the patient was placed in the supine position and a dorsiflexion osteotomy was performed at the first metatarsal. A 5-cm longitudinal skin incision was made from the proximal end of the shaft of the first metatarsal along the bony axis, and a 5-mm wide dorsal closed wedge osteotomy was performed on the first metatarsal (Figure 5), which was fixed with a locking plate.

**Figure 6:**
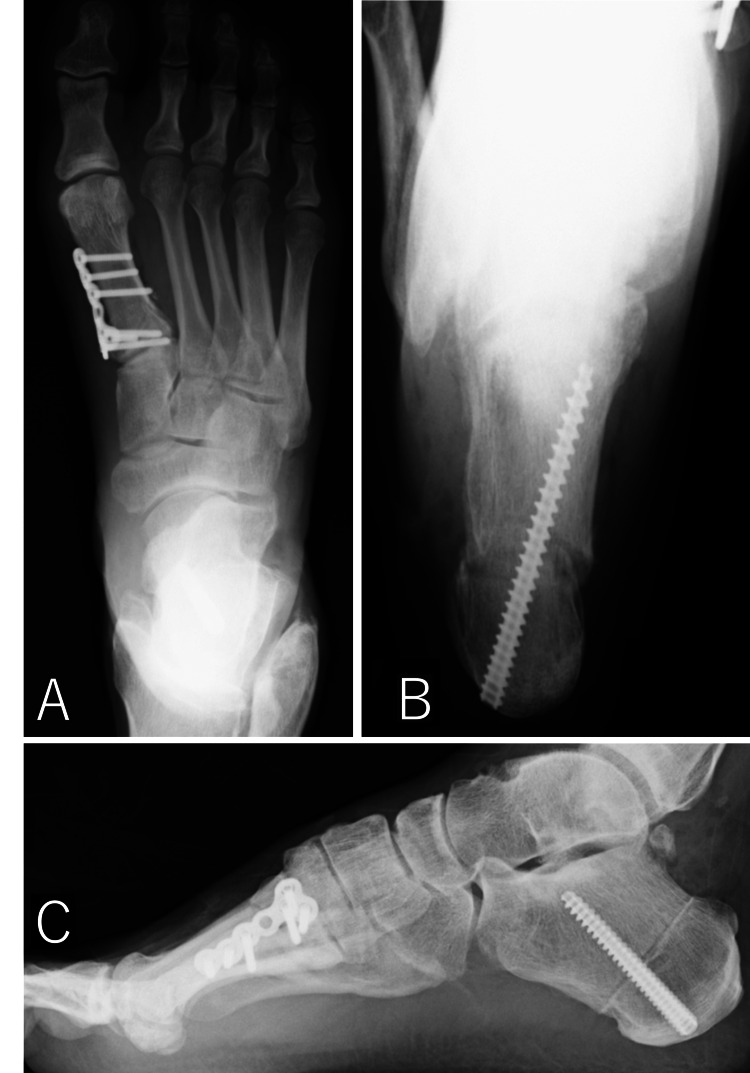
Postoperative plain radiographs (A) Dorsoplantar view. (B) Axial view of the calcaneus. (C) Lateral view.

This resulted in the flattening of the medial longitudinal arch and pronation of the ankle (Figure 6).

Postoperatively, he started range-of-motion training of the ankle joint and subtalar joint at one week. Then, he walked with a patella tendon-bearing orthosis and started foot loading exercises in a sitting position at 2 weeks, started 1/3rd partial weight-bearing at 6 weeks, 2/3rd partial weight-bearing at 8 weeks, and was full weight-bearing at 10 weeks.

Outcome and follow-up

**Figure 7:**
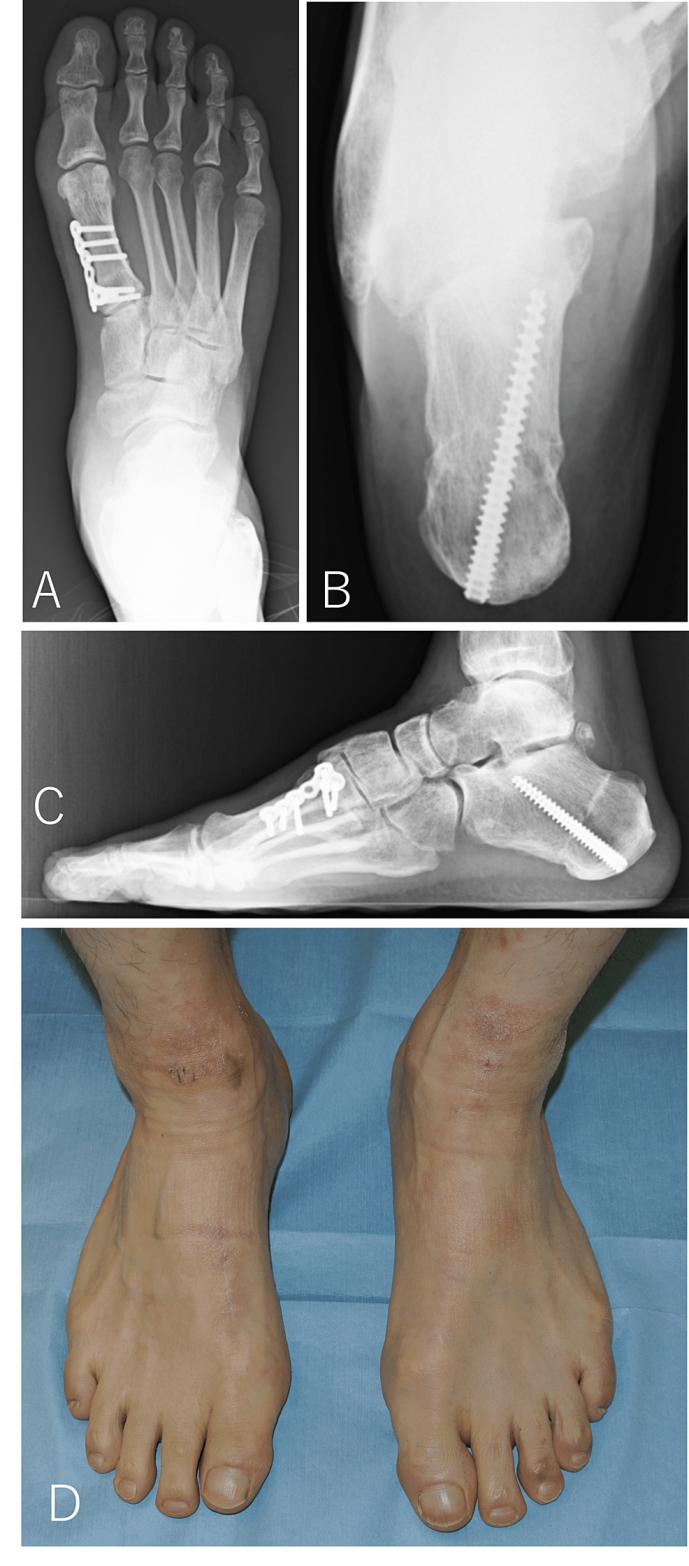
Plain radiographs and photograph one year after surgery (A) Dorsoplantar view. (B) Lateral view. (C) Axial view of the calcaneus. (D) Clinical photo.

At one year postoperatively, there was no particular pain, and the patient’s instability during walking had disappeared. He was able to resume recreational jogging. Meary's angle was corrected to 0°, the calcaneal pitch was corrected to 20°, and the hindfoot varus was improved (Figure 7).

**Figure 8:**
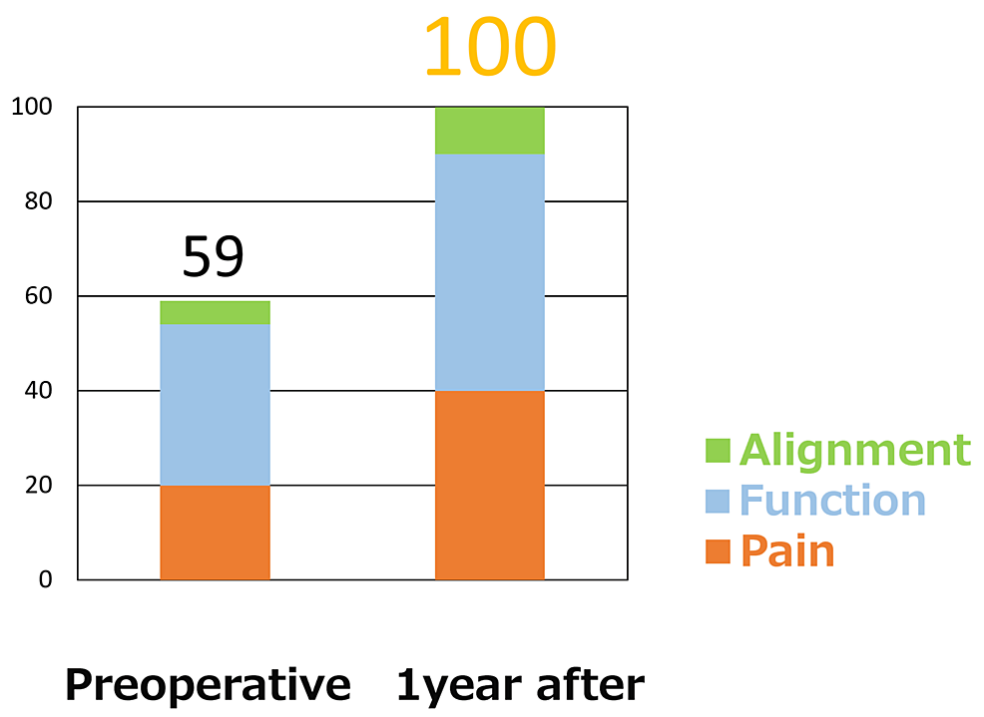
Changes in the JSSF (Japanese Society for Surgery of the Foot) ankle-hindfoot scale score

His Japanese Society of Surgery of the Foot ankle-hindfoot scale score [3,4] improved from 59 preoperatively to the maximum of 100 at one year after surgery (Figure 8).

**Figure 9:**
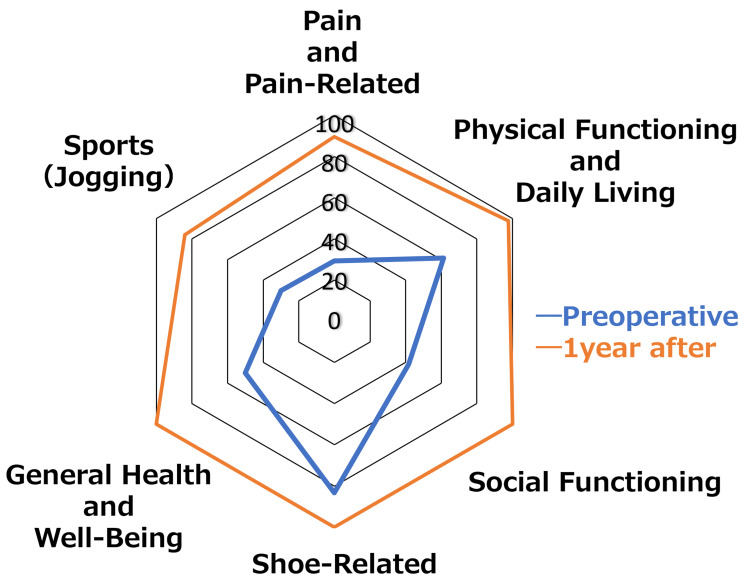
Results of the SAFE-Q (Self-Administered Foot Evaluation Questionnaire) The patient’s score was almost perfect for non-sports-related items and was 83.3 for sports-related items one year after surgery.

The patient’s responses to the Self-Administered Foot Evaluation Questionnaire [5] gave an almost perfect score for non-sports-related items and a score of 83.3 for sports-related items one year after surgery (Figure 9).

## Discussion

Peroneal tendinopathy was first reported by Meyer in 1924 [5]. The patient's chief complaint is pain along the peroneal tendon of the lateral side of the hindfoot, which increases with activity, especially on uneven or sloping terrain, puts stress on the subtalar joint, and is relieved with rest. Pain is induced by maximum inversion and by resistance exercises from the eversion position of the subtalar joint. A diagnosis of peroneal tendinopathy is made when the pain improves with an injection of a local anesthetic into the tendon sheath. Surgery can be considered in such cases when they are refractory to conservative treatment. Improvement is generally achieved by sheath incision and tendon repair. However, anatomical abnormalities, such as a giant peroneal tubercle [6], the presence of a fourth peroneal muscle [7], the presence of os peroneum [8], lateral ligament insufficiency [9], and pes cavus [1] have been identified to cause peroneal tendinopathy and should also be treated when present. Numerous peroneal tendinopathies due to giant osteophytes have been reported, and symptoms are exacerbated by changes in the trajectory of the tendons and increased tension. The cause of a giant peroneal tubercle has been reported to be a remnant of the embryonic period [10] or an increase in size over time [11]. In pes cavus, the medial longitudinal arch is often high, resulting in hindfoot varus, which causes traction at the attachment of the peroneal tubercle (peroneal trochlea) and increased tension on the peroneal tendon during walking. We believe that patients with pes cavus are more likely to develop a traction spur due to direct and indirect traction on the inferior peroneal retinaculum, as described above. In our case, the osteophyte was not massive and although it was not the direct cause of the peroneal tendon disorder, it was removed at the same time as the tendon sheath incision to decrease the tension on the tendon. Pes cavus is often accompanied by insufficiency of the lateral ligament, which is widely thought to require surgical repair [12,13]. In this case, the patient had supination sprains. We considered that this was due to the hindfoot varus caused by the pes cavus. This patient might have some degree of dysfunction of the lateral ligament. However, there have been several recent reports of good results without repair of the lateral ligament [14,15]. Therefore, we focused on correcting the bony alignment without repairing the ligaments. Dwyer osteotomy has been the method most widely for the correction of hindfoot varus [16], and a lateralizing calcaneal osteotomy is also frequently used nowadays [17]. The lateralizing calcaneal osteotomy has the advantages of being a simple surgical technique with adjustable movement and less reduction in tension on the Achilles tendon because there is no shortening of calcaneal length [17]. Furthermore, there is no evidence that osteotomies that are focused on the correction of valgus, such as the Dwyer osteotomy, are superior to lateralizing calcaneal osteotomy [18]. Therefore, we applied this technique in our case. Dorsiflexion osteotomy at the first metatarsal is useful in combination with calcaneal osteotomy when treating pes cavus [19]; in this case, calcaneal osteotomy alone was considered insufficient to correct the pes cavus, so an additional osteotomy was performed. The patient was satisfied with the results of his operation, reporting the resolution of his pain and sense of instability with no further sprains. We have two limitations. First, follow-up was completed after one year because the patient was satisfied with the results. Therefore, symptoms may appear in the future. Second, we didn’t evaluate the function of the lateral ligament. If the patient has a dysfunction of this ligament, ligament repair should be considered.

## Conclusions

The main complaint, in this case, was pain due to peroneal tendon disorder, but we considered that the pain was strongly influenced by the pes cavus. We believe that the treatment of the pes cavus, which was the main pathology, with lateralizing calcaneal osteotomy and first metatarsal dorsiflexion osteotomy, with the addition of peroneal tendon treatment as a tendon sheath incision and tendon repair, led to a good outcome. In the treatment of peroneal tendon disorders, it is important to consider the cause and select the appropriate surgical technique accordingly.
